# A Comparison of the Acute Effects of Percussion Massage Therapy and Static Stretching on Hamstring Elasticity

**DOI:** 10.4314/ejhs.v33i4.16

**Published:** 2023-07

**Authors:** Rumeysa Ateş, Pinar Yaşar, Ferdi Başkurt, Zeliha Başkurt, Sabriye Ercan

**Affiliations:** 1 Süleyman Demirel University, Faculty of Health Sciences, Department of Physiotheraphy and Rehabilitation, Isparta Turkey; 2 Süleyman Demirel University, Faculty of Medicine, Department of Sports Medicine, Isparta, Turkey

**Keywords:** Percussion massage, Hamstring flexibility, Static stretching, Acute effect

## Abstract

**Background:**

The effect of percussion massage on hamstring flexibility is unknown. This study aimed to investigate the acute effects of percussion massage on hamstring flexibility and to compare its effectiveness with static stretching.

**Methods:**

Fifty-four healthy individuals aged 18-25 years with at least 15 degrees of active knee extension were included in the study. The study was conducted between February and May 2022. The participants were randomly divided into 3 groups in this cross-randomization study as percussion massage (n=18), static stretching (n=18), and control (n=18). The Active Knee Extension test and the Sit and Reach test were used as evaluation parameters, and assessments were performed pre-intervention and 30 min post-intervention (acute).

**Results:**

In both percussion and stretching intervention groups, the range of motion (ROM) gain in the Active Knee Extension test was statistically significant (p<0.05) compared to the control group. Active knee extension angle gain was similar between percussion and stretching interventions (p>0.05). It was found that hamstring flexibility improved significantly in both percussion massage and static stretching groups (p<0.05). However, considering the last measurement and flexibility gain values, it was found that percussion massage and static stretching had similar acute effects on hamstring muscle flexibility (p>0.05).

**Conclusion:**

Percussion massage had an acute positive effect on hamstring flexibility and ROM, and it was as effective as static stretching. Therefore, percussion massage devices are recommended as part of pre-exercise in a structured warm-up for increase in joint range of motion and flexibility.

## Introduction

The hamstrings are a group of muscles made up of the semimembranosus, semitendinosus, and biceps femoris muscles (1). While these muscles are responsible for the movement of the hip and knee joints, they also control the alignment of the pelvis and spine (2). Flexing the knees in many activities or a sedentary life style may shorten the hamstring muscle, thus reduce flexibility (1). Hamstring shortening causes a decrease in posterior pelvic tilt and lumbar lordosis, leading to impaired postural alignment (2), and is considered one of the primary risk factors for hamstring strains (3). Decreased muscle flexibility can lead to changes in joint range of motion and damage in the musculoskeletal system (4). To prevent conditions such as postural changes, pain, and injury, it is important to maintain normal muscle length and regain the flexibility of shortened muscles (1,5).

Manual stretching exercises and mechanical applications are performed to increase muscle length (1). Stretching exercises can be listed as static, dynamic, ballistic, and proprioceptive neuromuscular facilitation (6). Static stretching is defined as gentle stretching applied to the muscle at the end point of the associated joint motion. With static passive stretching, the autogenic inhibition mechanism is activated, the viscoelastic properties of the tissue change, and the range of motion of the joint increases (3,7). In time-based examinations, it was concluded that the most effective application in gaining joint range of motion was 30 seconds of static stretching (4).

Another current method that is an alternative to stretching exercises in gaining flexibility is percussion massage therapy. Percussion massage therapy has gained popularity in the therapeutic and athletic communities over the past few years. Different manufacturers (eg., Theragun and, Hyperice) provide percussion devices for both self-massage and massage by a therapist. Such devices can vibrate at different frequencies up to 53 Hz. Depending on the tissue (i.e., soft tissue versus bony tissue), several attachment heads can be fixed to the devices so that local points can be massaged (8). This treatment combines elements of traditional massage and vibration therapy (8–11). With rhythmic contraction and relaxation, the Golgi tendon organ is stimulated, reducing abnormal muscle contraction and improving muscle length (12). Massage applied for 5 minutes with a hand-held percussion device provides an increase in ROM with the effect of myofascial relaxation (8).

To the best of our knowledge, there exists no study comparing the effectiveness of stretching exercise and percussion massage therapy, which has a positive effect on the flexibility of the hamstring muscle. How percussion massage therapy, which has become popular recently, leads to a change in joint range of motion by improving muscle flexibility compared to static stretching needs exploration. Our hypothesis was that percussion massage, similar to static stretching, would produce positive improvements in hamstring flexibility. Therefore, in our study, we aimed to compare the acute effect of static stretching exercise and percussion massage therapy on hamstring muscle flexibility.

## Materials and Methods

**Figure 1:**
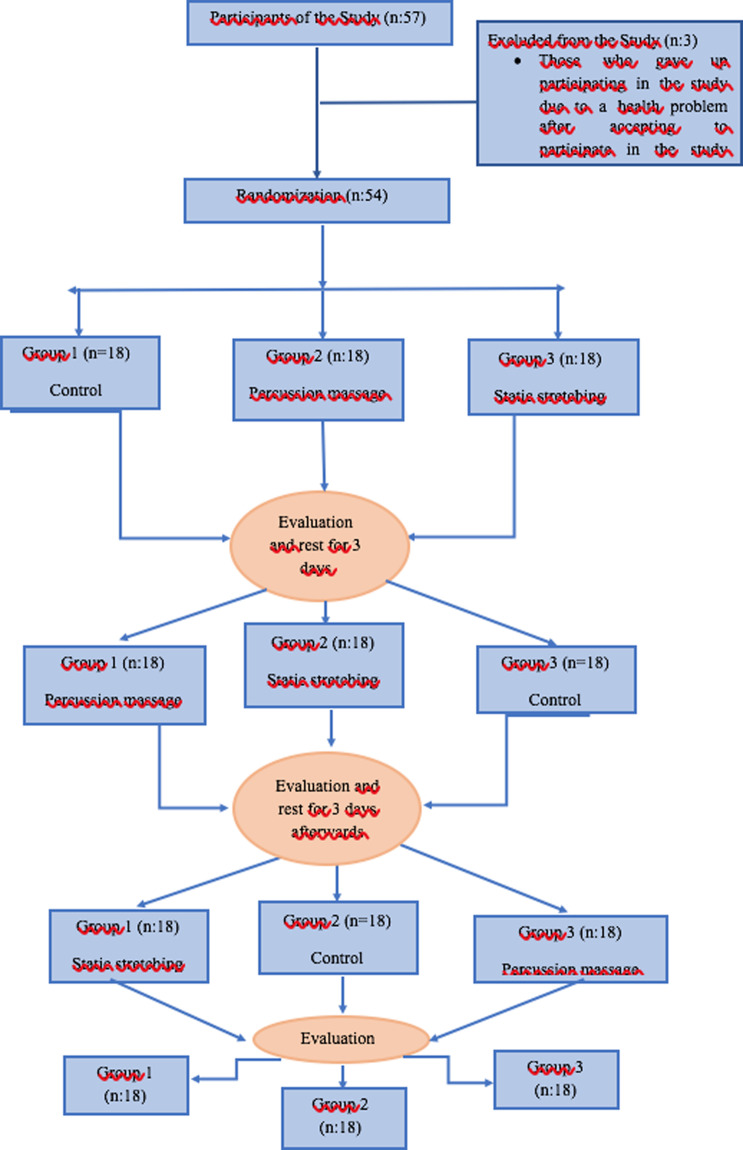
Flowchart of participants

The study, which was designed as “cross-randomization”, was carried out in the Rehabilitation Unit of the Süleyman Demirel University Faculty of Medicine, Department of Sports Medicine, between February and May 2022 (NCT05607914). The sample size of the study was calculated with the G Power 3.1 program using the study of Nakamura et al. (effect size d=0.705) as reference (13). A total of 54 healthy individuals aged 18-25 years with at least 15 degrees of active knee extension were included in the study. Those who had hamstring injury in the last two years, had neurological problems in the lower extremity and lumbar region, and those who participated in a similar study within the last year were excluded from the study. In accordance with the cross-randomization procedure, the participants were divided into 3 randomization groups, namely percussion massage, static stretching, and control (Figure 1). They were informed about the study and asked to sign a written consent form. The ethics committee approval of the study was obtained from Süleyman Demirel University Faculty of Medicine Clinical Research Ethics Committee (28/01/2022/72867572-050.01.04).

After the demographic characteristics of the participants were recorded, the dominant extremity was identified, and the baseline measurement before the application was performed. Dominant extremity was determined as the extremity preferred by individuals for hitting the ball (14). Then, the application was administered followed by the final measurement. The Active Knee Extension test (in the sitting position) and the Sit and Reach test were performed to evaluate hamstring flexibility. Before the tests, the participants were not allowed to have trials not to cause an increase in flexibility and angle.

**Active knee extension test**: In this test, which is used to evaluate hamstring flexibility in the sitting position, the participant was asked to sit on the edge of the stretcher and the hip-knee flexion angle was adjusted to 90°. For the measurement of active knee extension degree, a double-arm universal goniometer (Baseline Stainless Steel Goniometer; Fabrication Enterprises Inc., Elmsford, NY, USA) was placed on the lateral condyle of the femur and the participant was asked to actively extend the knee to full extension. The angle at the end point of the movement was recorded (12).

**Sit and reach test**: While sitting in a long sitting position with their knees straight and their bodies upright, the participants were asked to touch the assessment table with the soles of their bare feet. In this position, they were told to reach as far as they could reach on the desk with their elbows straight and palms facing the floor. The test was completed by the physiotherapist, who made the evaluation, by controlling the participant's maintaining the lower extremity position during the test, making sure that the heel did not leave the bench. By measuring the distance between the fingertip and the assesment table, those who could reach the table got “0” score, those who could reach beyond the table received (+) cm, and those who could not reach received (-) cm. The best value of two measurements was recorded (15).

**Application procedure**: Since no application was made in the control group, the participants were asked to rest for 30 minutes after the ‘initial measurements’ were administered. Then, the ‘final measurements’ were administered and recorded. To increase hamstring flexibility acutely, two different groups were created, namely the Static Stretching Exercise Group and the Percussion Massage Group.

For the static stretching exercise, the participant was asked to lie down in the supine position. The investigator passively flexed the dominant extremity hip to 90°, extending the knee to the maximum that could be tolerated. At the maximum point, the position was maintained for 30 seconds, and then the hamstring muscle was relaxed and rested for 30 seconds. A total of 5 minutes of application was completed by performing 5 repetitions with 30 seconds of static stretching and 30 seconds of rest in each period (8,16).

**Figure 2:**
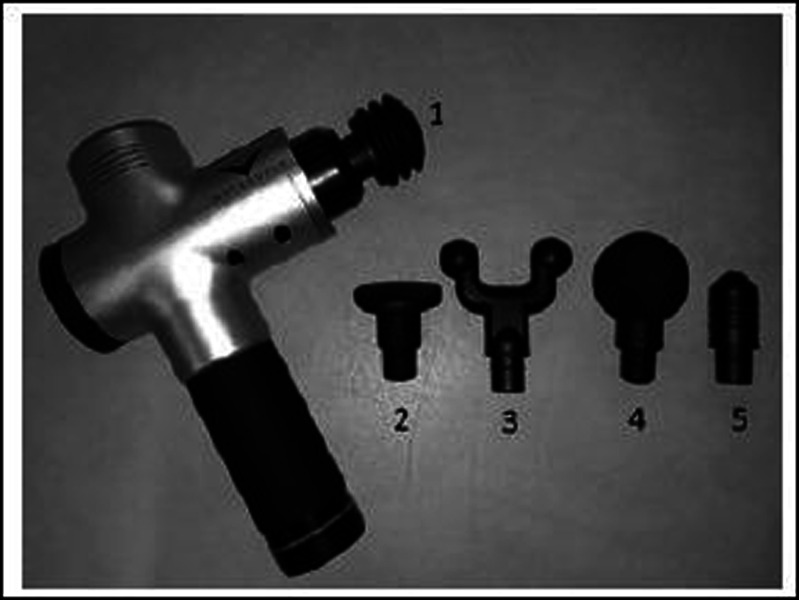
The handheld massage device (Hypervolt) with the different attachment heads which can be used. For this study was used head 4

Percussion massage was applied to the dominant hamstring muscle for 5 minutes at 53 Hz frequency with the “hard ball” head of the Hypervolt (Hyperice, California, US) device (Figure 2). Semitendinosus and semimembranosus muscles were focused in the first 2.5 minutes of the massage treatment, and the biceps femoris muscle in the second 2.5 minutes. In the first part of the application, the massage was started from the medial side of the muscle, paying attention to the equal amount of pressure applied throughout the massage. The massage was completed by moving the device from distal to proximal and from proximal to distal in a straight line within 20 seconds. In the last 2.5 minutes, the massage device was moved laterally at the distal end of the muscle, and the application was made from distal to proximal and then distally. Thus, the massage for each muscle started from the medial and ended laterally (8).

After the application, the participants were told to rest for 30 minutes, and at the end of 30 minutes, the measurements were repeated in all groups by the same physiotherapist. On the 4th and 7th days of the application, the people in the groups were interchanged and the whole procedure was repeated using the cross-randomization method. Subjects completed the three intervention conditions in different orders (Table 1).

**Table 1:** Applications during cross randomization

	1st randomization group (n=18)	2nd randomization group (n=18)	3rd randomization group (n=18)
1 day	Percussion massage	Static stretching	Control
4 days	Control	Percussion massage	Static stretching
7 days	Static stretching	Control	Percussion massage

**Statistical analysis**: Statistical analysis was performed with SPSS v.26.0 program (IBM Corp., Armonk, NY, USA). The suitability of the data for normal distribution was tested with the Kolmogorov Smirnov test and it was found that the groups were not suitable for normal distribution. The Friedman test was used to analyze the difference between groups. The Wilcoxon signed rank test was used to analyze the within-group difference. A p value of <0.05 was considered significant. Data are presented as mean±standard deviation, frequency (n), and percentage (%).

## Results

The study was conducted with a total of 54 participants with hamstring shortness. Demographic characteristics of the participants are given in Table 2.

**Table 2:** Demographic and descriptive data of participants

Variable and Category		Frequency (%)
Age (years) (mean±SD)		22.48±0.15
Gender	Female	42(77.8%)
	Male	12(22.2%)
Height (cm)		167.44±0.69
Body Weight (kg)		65.24±2.61
BMI (kg/m^2^)		22.49±0.45
Exercise Habit	Yes	7(13%)
	No	47(87%)
Dominant Extremity	Right	52(96.3)
	Left	2(3.7%)

BMI: Body Mass Index, Age and height variables are given as Mean±SD since they fit the normal distribution.

In both percussion and stretching groups, the range of motion (ROM) gain in the Active Knee Extension test was statistically significant compared to the control group (p<0.05). However, the active knee extension angle gain was similar between percussion and stretching groups (p>0.05) (Table 3).

**Table 3:** Active knee extension test results of the groups

Variable and category		Percussion	Stretching	Control	Intergroup p
	Beginning	73.41±0.41	73.31±0.44	73.65±0.41	0.782
	End	83.76±0.70^a^	83.48±0.69^a^	74.63±0.36^b^	0.0001
ROM (°)	ROM gain	10.29±0.53^a^	9.94±0.52^a^	0.98±0.14^b^	0.0001
	In-group p	0.0001	0.0001	0.0001	

There is a difference between groups denoted by different letters. ROM: Range of motion of the joint. According to the findings presented in Table 4, percussion massage and static stretching applications and hamstring flexibility improved significantly in both groups (p<0.05). However, when the last measurement and flexibility gain values were taken into account, the acute effects of percussion massage and static stretching on hamstring muscle flexibility were similar (p>0.05).

**Table 4:** Comparison of hamstring flexibility of the groups

Variable		Percussion	Stretching	Control	Intergroup p
	Beginning	18.54±1.12	18.13±1.11	18.42±0.07	0.491
	End	20.93±1.01^a^	20.67±1.01^a^	19.95±1.05^b^	0.007
Sit-Reach Test (cm)	Flexibility gain	2.41±0.29^a^	2.54±0.24^a^	1.54±0.09^b^	0.004
	In-group p	0.0001	0.0001	0.0001	

There is a difference between groups denoted by different letters

The elasticity gain was statistically significant between the baseline and final measurement of the control group (p<0.05). In addition, in the last measurement of the control application, there was a statistically significant difference between percussion massage and static stretching applications (p<0.05).

## Discussion

In this study, the effects of percussion massage and static stretching on hamstring flexibility were compared and it was observed that both applications provided an acute increase in active knee extension angle and hamstring flexibility. However, it was determined that percussion massage and static stretching were not superior to each other.

Percussion massage increases muscle length and flexibility via various physiological changes that occur during application (8,13,17,18). Percussion massage can change the fluid viscosity by causing pressure and friction on the applied muscle, skin, and fascia, thus leading to less resistance to movement. It was reported in the literature that percussion application can provide an increase in ROM by reducing the perception of pain (8). Therefore, it can be hypothesized that the changes in ROM following percussion massage can be explained by the reduction in muscle stiffness as well as the changes in pain perception (8). Another theory is that the Pacinian sensory receptors (mechanoreceptors) have a great influence on movement control and muscle activity, resulting from their response to local vibrations (18). Therefore, local vibrations applied with the percussion device can reduce the sensitivity to changes in muscle tension and muscle length, and an increase in ROM can be observed.

It has been reported in recent studies that percussion massage applied to the plantar flexor muscles of healthy individuals increases the range of motion of the ankle joint (8,13). In the systematic review conducted by Martin in 2021, 9 studies were reviewed and it was stated that percussion massage is an effective method for increasing lower extremity ROM(17). Jung et al. investigated the effect of local vibration massage on ROM in young patients with posterior shoulder tension and found an increase in the degree of internal rotation with shoulder adduction (18). Peloquin et al. evaluated the effect of massage application with a percussion massage gun on hamstring flexibility with the Sit and Reach test and determined that hamstring flexibility increased after 48 hours (19). In this study, which we conducted with healthy individuals with hamstring shortness, it was determined that percussion massage acutely increased knee ROM and hamstring flexibility in line with the literature.

Static stretching provides positive improvements in flexibility by helping to increase muscle length (20). Cini et al. examined the acute effect of passive static stretching applied at different times on hamstring flexibility and reported that static stretching increases hamstring flexibility regardless of time (21). In a study examining the effect of active and passive static stretching on hamstring ROM, it was seen that both applications increased ROM acutely, but the amounts of increase were similar (20,22). In our study, it was found that static stretching, which was applied similar to the literature, had acute positive effects on hamstring flexibility. Although the mechanisms responsible for the increase in muscle length after stretching are not fully understood (22), the theory that stress applied during stretching increases the number of serial sarcomere may explain this situation (23). On the other hand, the increase in viscoelasticity with the effect of stretching leads to a decrease in muscle and connective tissue stiffness, thus increasing the extensibility of the muscle (23).

To the best of our knowledge, the acute effects of percussion massage and static stretching on hamstring flexibility have not been studied before, and our study is the first study in the literature in this respect. The limitation of our study is that the effect of percussion massage on hamstring flexibility was examined only in the acute period, and its long-term results were not evaluated. It is recommended to examine the long-term effects in future studies. It is thought that our study will be a guide for future studies. Considering that percussion massage can be used as an alternative method to static stretching to prevent hamstring injuries and can increase flexibility in the chronic period, we recommend that future studies be conducted in this direction.

In conclusion, it was revealed that percussion massage is as effective as static stretching to increase hamstring flexibility and ROM in the acute period. In the light of the findings, it can be recommended to use percussion massage devices as a part of pre-exercise in a structured warm-up for increase in joint range of motion and flexibility. On the other hand, it would be appropriate to examine the effects of percussion massage on different variables such as muscle pain, muscle activation, reaction time, and strength in further studies.

## References

[1] Shamsi MB, Mirzaei M, Shahsavari S, Safari A, Saeb M (2020). Modeling the effect of static stretching and strengthening exercise in lengthened position on balance in low back pain subject with shortened hamstring: a randomized controlled clinical trial. BMC Musculoskeletal Disorders.

[2] Alshammari F, Alzoghbieh E, Abu Kabar M, Hawamdeh M (2019). A novel approach to improve hamstring flexibility: A single-blinded randomised clinical trial. The South African journal of physiotherapy.

[3] Kim DH, Lee JJ, You JSH (2018). Effects of instrument-assisted soft tissue mobilization technique on strength, knee joint passive stiffness, and pain threshold in hamstring shortness. Journal of back and musculoskeletal rehabilitation.

[4] Majeed A, Mansoor SR, Arif AB, Yasin MM, Wasim M, Naeem F (2021). Comparison of Static Stretching and Muscle Energy Techniques on Hamstring Tightness in Asymptomatic Females. Foundation University Journal of Rehabilitation Sciences.

[5] Okezue OC, Anamezie TH, Nene JJ, Okwudili JD (2020). Work-Related Musculoskeletal Disorders among Office Workers in Higher Education Institutions: A Cross-Sectional Study. Ethiopian journal of health sciences.

[6] Konrad A, Reiner MM, Thaller S, Tilp M (2019). The time course of muscle-tendon properties and function responses of a five-minute static stretching exercise. European journal of sport science.

[7] Mizuno T, Matsumoto M, Umemura Y (2013). Viscoelasticity of the muscle-tendon unit is returned more rapidly than range of motion after stretching. Scandinavian journal of medicine & science in sports.

[8] Konrad A, Glashüttner C, Reiner MM, Bernsteiner D, Tilp M (2020). The acute effects of a percussive massage treatment with a hypervolt device on plantar flexor muscles' range of motion and performance. Journal of Sports Science and Medicine.

[9] Cheatham SW, Baker RT, Behm DG, Stull K, Kolber MJ (2021). Mechanical percussion devices: A survey of practice patterns among healthcare professionals. International Journal of Sports Physical Therapy.

[10] Comeaux Z (2011). Dynamic fascial release and the role of mechanical/vibrational assist devices in manual therapies. Journal of Bodywork and Movement Therapies.

[11] Germann D, El Bouse A, Shnier J, Abdelkader N, Kazemi M, Germann D (2018). Effects of local vibration therapy on various performance parameters: a narrative literature review. The Journal of the Canadian Chiropractic Association.

[12] Lim JH, Park CB (2019). The immediate effects of foam roller with vibration on hamstring flexibility and jump performance in healthy adults. Journal of Exercise Rehabilitation.

[13] Nakamura M, Sato S, Kiyono R, Yoshida R, Murakami Y, Yasaka K (2021). Acute Effect of Vibration Roller With and Without Rolling on Various Parts of the Plantar Flexor Muscle. Frontiers in Physiology.

[14] Gabbard C, Hart S (1996). A question of foot dominance. The Journal of general psychology.

[15] Miyamoto N, Hirata K, Kimura N, Miyamoto-Mikami E (2018). Contributions of Hamstring Stiffness to Straight-Leg-Raise and Sit-and-Reach Test Scores. International journal of sports medicine.

[16] Davis DS, Ashby PE, McCale KL, McQuain JA, Wine JM (2005). The effectiveness of 3 stretching techniques on hamstring flexibility using consistent stretching parameters. Journal of strength and conditioning research.

[17] Martin J (2021). A critical evaluation of percussion massage gun devices as a rehabilitation tool focusing on lower limb mobility: A literature review. SportRxiv Preprints.

[18] Jung S, Ha S (2020). Effects of Local Vibration on Shoulder Horizontal Adduction and Internal Rotation Range of Motion in Subject with Posterior Shoulder Tightness. Journal of Musculoskeletal Science and Technology.

[19] Peloquin K, Barnhardt M, Behling G, Braun S (2022). The Immediate Effect of Percussion Myofascial Release Therapy on Hamstring Flexibility and Hip flexion Range of Motion Among Active Young Adults. International Journal of Research in Exercise Physiology.

[20] Nakao G, Taniguchi K, Katayose M (2018). Acute Effect of Active and Passive Static Stretching on Elastic Modulus of the Hamstrings. Sports Medicine International Open.

[21] Cini A, De Vasconcelos GS, Lima CS (2017). Acute effect of different time periods of passive static stretching on the hamstring flexibility. Journal of Back and Musculoskeletal Rehabilitation.

[22] Weppler CH, Magnusson SP (2010). Increasing muscle extensibility: a matter of increasing length or modifying sensation?. Physical therapy.

[23] Medeiros DM, Cini A, Sbruzzi G, Lima CS (2016). Influence of static stretching on hamstring flexibility in healthy young adults: Systematic review and meta-analysis. Physiotherapy Theory and Practice.

